# Effectiveness of an Intergenerational Service-Learning Program to Change Attitudes on Aging

**DOI:** 10.1093/geroni/igab046.1410

**Published:** 2021-12-17

**Authors:** Sara Bartlett, Sky Bergman, Phyllis Solomon, Zvi Gellis

**Affiliations:** 1 Cal Poly, San Luis Obispo, San Luis Obispo, California, United States; 2 University of Pennsylvania, Philadelphia, Pennsylvania, United States

## Abstract

This study evaluated the efficacy of a 10-hour intergenerational service-learning program administered to undergraduates to determine if it would increase knowledge about aging, improve attitudes about older adults, and reduce ageism more than a course with less service-learning activity. Making maximum impact on students in these areas in a short amount of time is particularly relevant in short, quarter-based university programs. A quasi-experimental design using a convenience sample compared pre-test and post-test scores between an experimental intervention (N=68) and a comparison (N=71) group on The Facts on Aging Quiz Multiple Choice version, Aging Semantic Differential, and Fabroni Scale on Ageism. Qualitative data via open-ended survey questions was also collected. The experimental intervention, the Lives Well Lived project, was based on a documentary film by the same name, which incorporates themes of successful aging. During the project students and older adults interviewed one another about living a life well lived, participated in a photo shoot, and created a Memoir for the older adult. The comparison group included two social visits to a congregate meal program. Results from multiple regression analysis showed that students in the intervention group had less ageist stereotypes and less negative bias about aging at post-test and qualitative data indicated they were more inclined to participate in intergenerational relationships in the future. Programs like this one that are longer and more relational may be useful for consideration in undergraduate gerontology courses in reducing ageism and promoting intergenerational relationships which benefit those of all ages.

